# RISK FACTORS OF LATENT TUBERCULOSIS INFECTION IN HEALTHCARE WORKERS AT HOSPITALS IN JEMBER CITY INDONESIA

**DOI:** 10.21010/ajid.v15i1.4

**Published:** 2020-12-14

**Authors:** Hamidah Retno Wardani, Ni Made Mertaniasih, Soedarsono Soedarsono

**Affiliations:** 1 Student of Master Program of Tropical Medicine, Faculty of Medicine, Universitas Airlangga, Jl. Mayjen. Prof. Dr. Moestopo No. 47, Surabaya 60131, Indonesia; 2 Department of Clinical Microbiology, Faculty of Medicine, Universitas Airlangga, Jl. Mayjen. Prof. Dr. Moestopo No. 47, Surabaya 60131, Indonesia; 3 Department of Pulmonology, Faculty of Medicine, Universitas Airlangga–Dr. Soetomo Hospital, Jl. Mayjen. Prof. Dr. Moestopo No. 47, Surabaya 60131, Indonesia

**Keywords:** Tuberculin skin test, Latent tuberculosis infection, Healthcare workers, Risk factors

## Abstract

**Background:**

Healthcare workers in Tuberculosis (TB) and non-TB units in hospitals have a high risk of experiencing Latent Tuberculosis Infection (LTBI), because of exposure to droplets containing *Mycobacterium tuberculosis*. This study aims to prove LTBI incidence and risk factors to healthcare workers at the hospital in Jember City.

**Material and Methods:**

a cross-sectional study, from January to March 2020 in two hospitals in Jember City. Healthcare workers in the TB care and non-TB care unit were examined using Tuberculin skin test (TST) with a cut off ≥ 10 mm for positive LTBI. Chest x-ray and clinical examination to rule out active TB and a standardized questionnaire were also used.

**Results:**

128 healthcare workers completed the questionnaires, clinical, tuberculin skin test (TST), and chest x-ray data. LTBI incidence of positive results 61.7% (n = 79). Contacts TB in the workplace (p value = 0.219; OR = 1.643; CI = 0.742-3.641) and a unit of work (p value = 0.102; OR = 0.760; CI = 0.559-1.031) has no relationship with LTBI. The profession (p value = 0.020; OR = 1.112; CI = 0.896-1.403), the duration of the work (p value = 0.039; OR = 2.984; CI = 1.067-8.342), and BCG immunization (p value =0.000; OR = 0.151; CI = 0.052-0.438) have important relationships with LTBI.

**Conclusion:**

TB infection with a high incidence, a risk of transmission to healthcare workers, and a relationship between occupational risk factors and LTBI among healthcare workers in Jember City, Indonesia have been established in this study.

## Introduction

The World Health Organization (WHO) estimated tuberculosis (TB) population worldwide as 10 million people in 2018. Indonesia ranks third with the highest incidence of TB after India and China. One alternative to prevent the spread, and to end tuberculosis epidemic is to screen individuals with LTBI as the key thing (WHO, 2019). Latent tuberculosis infection (LTBI) is a state of immune response resistant to the incentive of Mtbc infection, with no clinical manifestations of active TB (WHO, 2018). Individuals with LTBI do not have symptoms and are not contagious, but 5-10% are at risk of becoming active TB and being a source of infection (Houben & Dodd, 2016; Nasreen *et al.*, 2016; Sabri, *et al.*, 2019).

The highest prevalence of LTBI occurs in groups at risk of exposure such as healthcare workers. The prevalence of LTBI among nursing students (16.4%) and medical resident (37%) have been estimated (Kinikar *et al.*, 2019).

Reported LTBI in healthcare workers (15.7%) from 479 healthcare workers. LTBI prevalence was highest in physicians (27.8%), followed by HCWs without patient contact (23.45%), nurses (8.3%), and other HCWs in contact with patients (6.9%) (Kim *et al.*, 2018). Malaysia and Japan, which show an average prevalence of 10.6% and 9.9% respectively, while Taiwan has 14.5%, South Korea 17.2%, India 31% and China 33.6% (Almufty *et al.*, 2019). LTBI in healthcare workers at 34 primary health centers in Semarang City was 23.6% (Erawati & Andriany, 2020). LTBI in healthcare workers at 13 health centers in Surabaya City was 46.7% from 30 respondents (Andajani, 2019).

Healthcare workers play an important role in TB infection control in hospitals, but few hospitals pay attention to and check TB infection status among healthcare workers. We should carry out further studies to gather useful information to support and develop detailed guidelines for preventing and handling TB infection in hospitals through screening and treatment of LTBI in healthcare workers as a significant part of the TB infection control program in hospitals (Zhou *et al.*, 2014; Park, 2018). Tuberculin skin test (TST) and Interferon Gamma Release Assay (IGRA) are tests that can diagnose LTBI. But IGRA can raise costs if used to replace TST in other low and middle-income countries (CDC, 2014). TST is still a low-cost strategy undertaken for screening healthcare workers exposed to Mtb (Severo *et al.*, 2011). This study aims to analyze the risk points for the incidence of LTBI in healthcare workers at the Hospital in Jember City through clinical examinations, TST and chest x-rays.

## Materials and Methods

We organized this cross-sectional study in two hospitals in Jember City from January to March 2020. Inclusion criteria for the sample are healthcare workers in TB care units and non-TB care units; those who had no history of TB and TB treatment, and willingness to become a respondent. This was proven by filling in the informed consent after the explanation by the researcher. The exclusion criteria in this study were a previous TST reaction ≥ 15 mm, family in the home environment who have suffered from smear-positive TB, those that suffer from co-morbid and immunocompromised diseases (HIV, DM, CKD, cancer), those who received immunizations less than 1 month ago, those on immune suppressant drugs or corticosteroids, those who have experienced extensive burns or eczema, having had a viral infection (hepatitis) in the past month, pregnant subjects and those who suffer from active TB.

Stratified random sampling method was employed in the selection of the samples. A method of sampling for populations that have heterogeneous characteristics or the varied characteristics of population. Sampling was still carried out from the exposed group and the unexposed group. 128 respondents were selected, using the Lemeshow sample size formula adjusted for the total population in the hospital. The research variables studied were risk factors for LTBI in the workplace using clinical examinations, tuberculin skin test (TST) and chest x-rays got from secondary data.

Risk factors include TB contact history, profession, work unit, length of work in a hospital, duration of working hours per day, use of N95 masks, and history of BCG immunization obtained from primary data. Each selected health worker filled out a standardized questionnaire. The questionnaire contains questions about initial identity and risk factors for health workers such as demographic information, family medical history, and history of TB with a positive smear, respondent’s medical history, and current work history and history of BCG vaccine. 

The single-step TST uses 10 international units (IU; 0.1 ml) of tuberculin (purified protein derivative). We performed TST using the Mantoux method by experienced personnel by injecting tuberculin intracutaneously with a distance of 2-3 inches from the crease of the elbow on the upper surface. Respondents returned 48-72 hours after their TST for results, and a specialist confirmed the outcome of the test result. We measured the horizontal diameter of the induration using a standardized ruler, cutoff and an experienced specialist read the results. LTBI was determined using TST induration with a cut off point ≥ 10 mm for a positive TST. All respondents also went through clinical evaluation and chest x-ray examination confirmed by a radiology specialist to rule out active TB. Consent information sheet signatures were taken from the respondents and we guarded the information with responsibility. All respondents had information about the results of the LTBI examination and could immediately consult the doctor responsible for further management.

We analyzed data using Statistical Product and Service Solutions (SPSS) for windows version 25.0. We displayed each variable in the form of frequency and percentage and analyzed the relationship between risk factors and LTBI incidence using the chi-square test with a significant relationship of the p-value<0.05. Multivariate analysis using binary logistic regression analysis. The independent variable with a value of p≤0.25 in bivariate analysis can be included in the model, and we can determine its probability. We coded all data obtained from the respondents and kept them confidential and stored properly. This study received ethical permission from the Ethics Review Board of Jember Paru Hospital, East Java Province, 002 / RSP / KEPK / 2020. TST in this study has gone through a clinical trial site , in accordance with the previous clinical trial site using PPD RT 23. Link internet: https://www.clinicaltrials.gov/ct2/show/NCT01241188

## Result

Data were obtained from 128 respondents and all of them completed the interview. All respondents went through a TST examination with a cutoff ≥ 10 mm for a positive TST result. After carrying out the TST test, all respondents conducted a chest x-ray examination to rule out active TB. The female gender respondents were more (66/128) or 51.6% and they dominated the age range 30-39 years (65%). Sixty eight per cent (68%) of the respondents had a normal BMI (18.5-25). Respondents do not smoke (79.7%) and have a tertiary education (84.6%). We present the characteristics of the respondents in table 1.

**Table 1:** Demographic characteristics of healthcare workers at the hospital

Variable	LTBI positive (61.7%)		LTBI negative (38.3%)		Total (100%)		P-value

	n	%	n	%	n	%	
TB contacts at work							
- Yes	61	77.2	33	67.3	94	73.4	0.219
- Not	18	22.8	16	32.7	34	26.6	
Type of Profession							
- Doctor	0	0	3	6.1	3	2.3	0.020
- Nurse	53	67.1	35	71.4	88	68.8
- Microscopic laboratory personnel	5	6.3	0	0	5	3.9
- Radiology laboratory personnel	1	1.3	0	0	1	8
- Pharmacy	3	3.8	5	10.2	8	6.2
- Admin	6	7.6	1	2.0	7	5.5
- Cleaning service	7	8.9	2	4.1	9	7.0
- Other	4	5.1	3	6.1	7	5.5	
Work unit							
- TB treatment	20	25.3	16	32.7	36	28.1	0.102
- Emergency	4	5.1	4	8.2	8	6.2
- Non TB Treatment	31	39.2	21	42.9	52	40.6
- Pharmacy	3	3.8	4	8.2	7	5.5
- Nutrition	3	3.8	1	2.0	4	3.1
- Micro / radiology laboratory	6	7.6	0	0	6	4.7
- Other	12	15.2	3	6.1	15		11.7
Length of work							
- <10 years	47	59.5	33	67.3	80	62.5	0.372
- ≥ 10 years	32	40.5	16	32.7	48	37.5	
Duration of work							
- <8 hours	24	30.4	7	14.3	31	24.2	0.039
- ≥ 8 hours	55	69.6	42	85.7	97	75.8	
Use of N95 masks							
- Always	36	45.6	24	49.0	60	46.9	0.928
- Sometimes	28	35.4	16	32.7	44	34.4	
- Never	15	19.0	9	18.4	24	18.8	
History of BCG Immunization							
- Yes	43	54.4	44	89.8	87	68.0	0.000
- Not	36	45.6	5	10.2	41	32.0	

The positive incidence of TST among healthcare workers was 79 (61.7%) from 128 respondents. Table 2 shows that most healthcare workers had a history of TB contact in the workplace (73.4%, p-value = 0.219; OR = 1.643, CI = 0.742-3.641) had no relationship with the occurrence of LTBI. The profession which is mostly dominated by nurses (68.8%) has a significant relationship with the occurrence of LTBI in healthcare workers (p-value = 0.020; OR = 1.112; CI = 0.896-1.403). Duration of work (p-value = 0.039; OR = 2.984; CI = 1.067-8.342) and history of BCG immunization (p-value = 0.000; OR = 0.151; CI = 0.052-0.438) has a relationship with the occurrence of LTBI in healthcare workers. There was no significant relationship in the work unit (p-value = 0.102; OR = 0.760; CI = 0.559-1.031), length of work (p-value = 0.372) and the habit of using masks in the hospital (p-value = 0.928) (Table 2).

**Table 2:** Bivariate analysis of factors associated with LTBI incidence in healthcare workers BMI Body Mass Index, LTBI Latent Tuberculosis Infection

Variable	LTBI positive (61.7%)		LTBI negative (38.3%)		Total (100%)	

	n	%	n	%	n	%
Gender						
- Male	42	53.2	20	40.8	62	48.4
- Women	37	46.8	29	59.2	66	51.6
Age						
- 20-29	18	22.8	17	34.7	35	27.3
- 30-39	41	51.9	24	49	65	50.8
- 40-49	14	17.7	5	10.2	19	14.8
- > 50	6	7.6	3	6.1	9	7.0
BMI						
- ≤ 18.5	4	5.1	1	2.0	5	3.9
- 18.5-25	55	69.6	32	65.3	87	68.0
- > 25	20	25.3	16	32.7	36	28.1
Smoking habit						
- Yes	19	24.1	7	14.3	26	20.3
- Not	60	75.9	42	85.7	102	79.7

### TB Tuberculosis, LTBI Latent Tuberculosis Infection, BCG Bacillus Calmette Guerin

The multivariate analysis in this study showed that healthcare workers with a history of TB contact in the workplace had a 1.64 times greater risk, and healthcare workers who had a work duration of ≥ 8 hours had a 2.98 times greater risk for LTBI (Table 3).

**Table 3:** Multivariate analysis associated with positive LTBI in healthcare workers

Variable	OR (95% Coefficient Interval)
TB contacts at work	1.643 (0.742-3.641)
Type of profession	1.112 (0.896-1.403)
Work unit	0.760 (0.559-1.031)
Duration of work	2.984 (1.067-8.342)
BCG immunization	0.151 (0.052-0.438)

OR Odd ratio, BCG Bacillus Calmette Guerin

## Discussion

Indonesia comprises 34 provinces. One of the most populated islands is Java (Erawati and Andriany, 2019).

Java Island comprises 6 provinces, one of them is East Java. The data showed that East Java ranks first as a province with the highest TB incidence in Indonesia. One city in East Java with the second-highest incidence in East Java is the City of Jember. Data from the Jember District Health Office shows that in 2017 the rate of tuberculosis in Jember was 3,479 cases (https://www.kemkes.go.id/resources/download/info-terkini/materi%20pra%20rakerkesnas%202018/04_%20Paparan%20Kadinkes%20Kab%20Jember.pdf).

Healthcare workers have a high risk of being exposed to tuberculosis in the workplace. LTBI screening of healthcare workers is an important step to reduce incidence. Based on the data obtained from this study, the prevalence of LTBI is higher than the study conducted in Peru (56.2%) from 190 respondents (Sedamano *et al.*, 2020). The prevalence of LTBI in this study was higher when compared to studies conducted in China. The TST-positive prevalence was 58% of 127 in the infectious hospital and 33.9% of 105 in the non-TB hospital (Zhou *et al.*, 2014).

Differences in the characteristics and burden of TB can cause these differences in each city. Based on demographic data, the gender with the more positive LTBI results was the male gender, 42 (53.2%). This is in agreement with earlier reports in 2012 and 2015 that the proportion of LTBI was higher in adult males (He *et al.*,2012 ; Ting *et al.*, 2014; He *et al.*, 2015). Another study reported that the prevalence of LTBI was more common in women with a positive number of TST 64 of 198 respondents (Janagond *et al.*, 2017).

The profile on age and positive LTBI results were dominated by the ages of 30-39 years (41/128 or 65%). A study reported that ages 35-44 and 45-60 years have a very significant relationship with the episode of LTBI (Sabri *et al.*, 2019). Another study still reported that healthcare workers aged 30-39, 40-49 years and ≥ 50 years have a greater risk of developing LTBI with a percentage of 33.9%, 44.2%, and 46.3% (Chen *et al.*, 2018). The Elderly is a condition in which the immune system will go through various changes and experience a decline (Simon *et al.*, 2015). One indicator is the characteristic changes in T cells which play an important role in defending and fighting microorganisms such as viruses and bacteria (Deng *et al.*, 2019). This condition can have the impact of increasing susceptibility to TB infection (Erawati & Andriany, 2020). 

Our results reported that 4 (5.1%) respondents with low BMI and 20 (25.3%) respondents with a BMI more than normal had positive LTBI. A study explains that BMI affects immunity and the important role of chemokine as the basis of naïve T cells, effectors, and differentiation of memory T cells and T cell work (Griffth *et al.*, 2014). 

Our study reports that profession is a risk component for LTBI. The risk factors for the profession with the highest positive TST result occurred in nurses. Here, the proportion of nurses is more than other health workers. Another study reported that respondents in their research had the most positive QFT-G results on physicians and nurses, amounting to 20 (20%) and 12 (7.5%) (Bukhary *et al.*, 2018). Also reported that the most positive results for LTBI were in the nursing profession 19 respondents. This is because nurses are the professionals that most often have contacts with patients, including active TB patients (Kumar *et al.*, 2019).

Another factor that affects LTBI is the duration of work for health workers. Our study reported that work duration of ≥ 8 hours (69.6%) had a higher risk of developing LTBI. Another study reported that the long duration of work in the TB care unit was a risk factor for LTBI (Park, 2018). Besides, the effectiveness of TB transmission influences the duration of action, including factors associated with index cases and TB contacts, the number of infectious dosages (one infection is present in 340 m^3^ of air) in the air leading to effective transmission. The number of bacteria found in droplets also affects transmission and transmission to other individuals (Fennelly& Jonez-Lopez, 2015; Migliori *et al.*, 2019).

We also found that TB contacts at work were at high risk for LTBI. The Rotterdam study illustrated the effect of proximity to TB transmission in that 35% of close contact index cases had positive results on their sputum compared to 10% of casual contacts (Migliori *et al.*, 2019). Earlier research stated that the prevalence of LTBI among healthcare workers still occurred in officers who had contact with TB patients (89.2%). In line with what was stated by the CDC that working hours, working conditions, and close contact with patients at high risk were risk factors for experiencing LTBI (CDC, 2014). The work unit in our study was a risk component for positive LTBI results for healthcare workers in TB care rooms **(**He *et al.*, 2012). This is in agreement with a previous report that stated that working in a unit related to TB patients can increase the risk of exposure to healthcare workers (Zhang *et al.*, 2013). 

Other studies show that the experience of being exposed to TB patients who have not been treated is greater in frequency for healthcare workers who work in TB care units compared to healthcare workers who work in non-TB care units, 78.8%, and 61.9%. TB patients are very infectious before starting treatment. Healthcare workers who work in TB care units are more at risk of being infected with TB compared to healthcare workers who work in non-TB care units (Park, 2018). 

Healthcare workers do personal protective equipment such as N95 masks as infection prevention. But, in this study, we found that many healthcare workers who always use N95 masks had positive TST results, 36 (45.6%) respondents. Another study reported that respondents who did not comply with PPE obtained positive results on sputum AFB. Another study reported that TST positivity was more prevalent in respondents who did not use N95 masks 77.4% of 153 respondents (Anwar *et al.*, 2019). Using N95 masks must be accompanied by compliance and procedures. The purpose of the N95 mask personal protective equipment is to reduce the exposure of health workers in the work environment with contaminated air and protect from droplet nuclei (Migliori *et al.*, 2019). Other factors that can affect the positivity of LTBI screening for respondents who always use N95 are adherence. Lack of compliance in the management of, control and prevention of infection in droplet transmission along with management of the placement of patients in the treatment room can affect the transmission and transmission process to health workers (Douedi & Douedi, 2019). 

Another influencing factor is respondents with a history of BCG vaccine, who also had positive TST results 32% of 162 respondents (Janagond *et al.*, 2017). A study examining the sensitivity and specificity of TST in healthcare workers in countries with a high TB burden recommended using a cutoff ≥ 10 mm, which BCG program (CDC, 2014).

In addition, several studies reported a review of randomized controlled trials showing BCG to be effective in protecting against LTBI for up to 10 years (Chen *et al.*, 2015).

Although the incidence of TB is decreasing in many countries, there is still a risk of transmission in hospitals because of delayed diagnosis and inadequate facilities for infection control programs, which can cause an increasing proportion of healthcare workers to be infected. Although the management of active TB screening and searches is rare among healthcare workers in hospitals in the City of Jember, data on the prevalence of LTBI is the major concern for prevention and reduction in the incidence of active TB, especially among healthcare workers.

The limitations of this study are the limited independent variables studied, LTBI is an infectious problem that arises because it is linked to many risk factors that are not only caused by work but a history of the previous contact either at work or at home. Some risk factors not examined here were family history of TB contacts and neighbor history of TB contacts.

## Conclusions

This study revealed that prevalence of LTBI (61.7%) among healthcare workers was more affected by risk factors such as a history of TB contact in the workplace of profession and duration of work ≥ 8 hours. In this epidemiological context, screening and searching for TB and LTBI among hospital healthcare workers is necessary for TB control efforts.

**List of Abbreviations:**
BCG: – Bacillus Calmette-Guerin
BMI: – Body Mass Index
CFU: – Colony Forming Unity
IGRA: – Interferon Gamma Release Assay
LTBI: – Latent Tuberculosis Infection
MHC: – Major Histocompatibility Complex
mm: – millimeter
OR: – Odd Ratio
TB: - Tuberculosis
TST: – Tuberculin Skin Test
WHO: - World Health Organization

## References

[1] Almufty H. B, Abdulrahman I. S, Merza M. A (2019). Latent tuberculosis infection among healthcare workers in Duhok Province:from screening to prophylactic treatment. Tropical Medicine and Infectious Disease.

[2] Andajani S (2019). Determinant of latent pulmonary tuberculosis incidence among health workers in community health centers in Surabaya, Indonesia. Folia Medica Indonesia.

[3] Anwar M. M, Ahmed D. M, Elareed H. R, Abdel-Latif R. A. R, Sheemy M. S, Kamer N. M, Mohammed M. F (2019). Screening for latent tuberculosis among healthcare workers in an egyptian hospital using tuberculin skin test and quantiFERON-TB gold in-tube test. Indian Journal of Occupational and Enviromental Medicine.

[4] Bukhary Z. A, Amer S. M, Emara M. M, Abdalla M. E, Ali S. A (2018). Screening of latent tuberculosis infection among health care workers working in hajj pilgrimage area in saudi arabia, using interferon gamma release assay and tuberculin skin test. Annals of Saudi Medicine.

[5] Centers of Disease Control and Prevention. (2014) Tests for TB infection:tuberculin skin test (TST).

[6] Chen B, Gu H, Wang X, Wamh F, Peng Y, Ge E, Upshur R, Dai R, Wei X, Jiang J (2018). Prevalence and determinants of latent tuberculosis infection among frontline tuberculosis healthcare workers in southeastern China:A multilevel analysis by individuals and health facilities. International Journal of Infectious Disease.

[7] Chen C, Zhu T, Wang Z, Peng H, Kong W, Zhou Y, Shao Y, Zhu L, Lu W (2015). High latent TB infection rate and associated risk factor in the Eastern China of low TB incidence. PLoS ONE.

[8] Deng Y, Liu Y, Li Y, Jing H, Wang Y, Li X, Xu L (2019). Isolation measures and protection awareness are significant for TB laten:a cross-sectional study on T-SPOT.TB among health care workers in China. Epidemiology of Infection.

[9] Douedi S, Douedi H (2019). Precautions, Bloodborne, Contact, and Droplet.

[10] Erawati M, Andriany M (2020). The prevalence and demographic risk factors for latent tuberculosis infection (LTBI) among healthcare workers in Semarang, Indonesia. Journal of Multidisciplinary Healthcare.

[11] Fennelly K. P, Jonez-Lopez E. C (2015). Quantity and quality of inhaled dose predicts immunopayhology in tuberculosis. Frontiers in Immunology.

[12] Griffth J. W, Sokol C. L, Luster A. D (2014). Chemokines and chemokine receptors:positioning cells for host defense and immunity. Annu Rev Immunology.

[13] He G. X, Wang L. X, Chai S. J, Klena J. D, Cheng S. M, Ren Y. L, Ren P. L, Gao F, Li Y Y, He G M, Li J B, Rao C, Varma J. K (2012). Risk factors associated with tuberculosis infection among health care workers in Inner Mongolia, China. International Journal Tuberculosis Lung Disease.

[14] He G, Li Y, Zhao F, Wang L, Cheng S, Guo H, Klena J. D, Fan H, Gao F, Gao F, Han G, Pen L, Song Y, Xiong Y, Geng M, Hou Y, He G, Li J, Guo S, Yang J, Yan D, Wang Y, Gao H, An J, Duan X, Wu C, Duan F, Hu D, Lu K, Zhao Y, Rao C. Y, Wang Y (2015). The prevalence and incidence of latent tuberculosis infection and its associated factors among village doctors in china. PLoS One.

[15] Houben M. G, Dodd P. J (2016). The global burden of latent tuberculosis infection:a re-estimation using mathematical modelling. PLoS ONE.

[16] Janagond A. B, Gamesan V, Kumar S. V, Ramesh A, Anand P, Mariappan M (2017). Screening of health-care workers for latent tuberculosis infection in a tertiary care hospital. The International Journal of Mycobacteriology.

[17] Jember District Health Office. (2017) Regional Support In The Accelartion Of TB Elimination Program In Jember District–East Java.

[18] Kim S, Choi H, Jang Y J, Park S, Lee H (2018). Prevalence of and factors related to latent tuberculous infection among all employees in a referral hospital. International Journal Tuberculosis Lung Disease.

[19] Kinikar A, Chandanwale A, Kadam D, Joshi S, Basavaraj A, Pardeshi G, Girish S, Shelke S, DeLuca A, Dhumai G, Golub J, Lokhande N, Gupte N, Gupta A, Bollinger R, Mave V (2019). High risk for latent tuberculosis infection among medical residents and nursing students in India. PLoS ONE.

[20] Kumar M. G, Joseph B, Gound B. R, Joseph M, Rajitna M (2019). Risk of tuberculosis infection among healthcare workers in a tertiary care hospital in Bengaluru city. Indian Journal of Accupational &Environmental Medicine.

[21] Migliori G. B, Nardell E, Yedilbayev A, D'Ambrosio L, Centis R, Tadolini M, Boom M. V. D, Ehsani S, Sotgiu G, Dara M (2019). Reducing tuberculosis transmission:a consensus document from the world health organization regional office for Europe. European Respiratory Journal.

[22] Nasreen S, Shokoohi M, Malvankar-Mehta M. S (2016). Prevalence of latent tuberculosis among health care workers in high burden countries:a systematic review and meta-analysis. PLoS ONE 2016.

[23] Park J. S (2018). The prevalence and risk factors of latent tuberculosis infection among health care workers working in a tertiary hospital in South Korea. A Journal of Clinical Medicine.

[24] Sabri A, Quistrebert J, Amrani H. J, Abid A, Zegmot A, Ghorfi I. A, Souchi H, Boucaid A, Benali A, Abilkassem R, Kmari M, Hassani A, Lahcen B, Siah S, Schurr E, Boisson-Duppuls S, Casanova JL, Lahlou A, Laatiris Abdelkader, Louzi Lhoussain, Ouarssani A, Bourazza A, Aouragh A, Mustapha B, Messaoudi N, Agader A, Cobat A, Abel L, El-Baghdadi J (2019). Prevalence and risk factors for latent tuberculosis infection among healthcare workers in Morocco. PLoS ONE.

[25] Sedamano J, Schwalb A, Cachay R, Zamudio C, Ugarte-Gil C, Soto-Cabezas G, Munayco C. V, Seas C (2020). Prevalence of positive TST among healthcare workers in high-burden TB setting in Peru. BMC Public Health.

[26] Severo K. G. P, Oliveira Jd. S, Carneiro M, Valim A. R. M, Krummenauer E. C, Possuelo L. G (2011). Latent tuberculosis in nursing professional of a Brazilian hospital. Journal of Occupational Medicine and Toxicology.

[27] Simon A. K, Hollander G. A, McMichael A (2015). Evolution of the immune system in humans from infancy to old age. Proc R Soc B.

[28] Ting W. Y, Huang S. F, Lee M. C, Lin Y. Y, Lee Y. C, Feng J. Y, Su W. J (2014). Gender disparities in latent tuberculosis infection high-risk individuals:A cross-sectional study. PLoS ONE.

[29] World Health Organization (2018). Latent tuberculosis infection:Updated and consolidated guidelines for programmatic management.

[30] World Health Organization (2019). Latent tuberculosis infection:Updated and consolidated guidelines for programmatic management. Background document on the 2019 revision.

[31] World Health Organization (2019). Programmatic management of latent tuberculosis infection, South-east Asia regional action plan.

[32] Zhang X, Jia H, Liu F, Pan L, Xing A, Gu S, Du B, Sun Q, Wei R, Zhang Z (2013). Prevalence and risk factors for latent tuberculosis infection among health care workers in China:a cross-sectional study. PLoS ONE.

[33] Zhou F, Zhang L, Gao L, Hao Y, Zhao X, Liu J, Lu J, Li X, Yang Y, Chen J, Deng Y (2014). Latent tuberculosis infection and occupational protection among health care workers in two types of public hospital in China. PLoS ONE.

